# Chinese herbal medicine TangBi Formula treatment of patients with type 2 diabetic distal symmetric polyneuropathy disease: study protocol for a randomized controlled trial

**DOI:** 10.1186/s13063-017-2345-1

**Published:** 2017-12-29

**Authors:** De Jin, Wen-jing Huang, Xiang Meng, Fan Yang, Yu-jiao Zheng, Qi Bao, Mei-zhen Zhang, Ya-nan Yang, Qing Ni, Feng-mei Lian, Xiao-lin Tong

**Affiliations:** 1 Guang’anmen Hospital, China Academy of Traditional Chinese Medical Sciences, No. 5, Bei xian ge Xicheng District, Beijing, 100053 China; 2 Beijing University of Chinese Medicine, No. 11, Bei San Huan Dong Lu, Chaoyang District, Beijing, 100029 China

**Keywords:** Diabetic distal symmetric polyneuropathy, Traditional Chinese medicine, Treatment, Clinical trials

## Abstract

**Background:**

Diabetic distal symmetric polyneuropathy (DSPN) is one of the most common microvascular complications of diabetes mellitus, and it has become a major public health problem worldwide because of its high and increasing prevalence, morbidity, and disability rate. The current medications for DSPN are not entirely satisfactory. Preliminary studies indicated that the Chinese herbal TangBi Formula may alleviate signs and symptoms and improve the velocity of nerve conduction in patients with DSPN. This study was designed to determine if Chinese herbal medicine used in combination with conventional treatment is more effective than conventional treatment alone.

**Methods/design:**

We are conducting a multicenter, placebo-controlled, double-blind, randomized, controlled clinical trial as a means of assessing the therapeutic effects of traditional Chinese medicine (TCM) treatment. A total of 188 patients will be randomized in a 1:1 ratio to a treatment group (TangBi Formula plus mecobalamin) and a control group (placebo plus mecobalamin). The test period lasts 6 months, during which all of the patients will be given standard medical care as recommended by established guidelines. The primary outcome will be development of differences in changes in clinical symptoms and signs in patients and changes in Michigan Diabetic Neuropathy Score (MDNS) between the two groups before and after treatment. The secondary outcome will be changes in nerve conduction velocity and in single clinical signs and symptoms. Safety assessments and adverse events will also be evaluated.

**Discussion:**

We postulate that patients with DSPN will benefit from therapy that includes TCM. If successful, this work will provide an evidence-based complementary therapeutic approach for treatment of DSPN.

**Trial registration:**

ClinicalTrials.gov, NCT03010241. Registered on 2 January 2017

**Electronic supplementary material:**

The online version of this article (doi:10.1186/s13063-017-2345-1) contains supplementary material, which is available to authorized users.

## Background

The latest figures from the International Diabetes Federation indicate that as of 2015, more than 415 million people worldwide have diabetes, and this number is expected to increase to 642 million by 2040. China has the largest number of people with diabetes (109.6 million) [1]. An epidemiological survey done in China shows that, among adults in China, the estimated overall prevalence of diabetes is 10.9% [2]. Diabetic individuals have higher rates of premature death, functional disability, and coexisting illnesses than their healthy counterparts. During its clinical course, diabetes may be accompanied by several other microvascular and macrovascular complications [3]. Some of the most serious and deleterious complications of diabetes, such as foot ulcers, are associated with diabetic neuropathy. Distal symmetric polyneuropathy (DSPN) is the most common neuropathic complication of diabetes. Up to 50% of patients with neuropathy experience symptoms, including burning pain, stabbing sensations, paresthesia, hyperesthesia, and deep aching pain, which typically becomes worse at night [4–6]. The end-stage complications of neuropathy, such as foot ulceration and amputation, are associated with a dramatic increase in health care costs and a decrease in quality of life [7–9]. Painful neuropathic symptoms can interfere with sleep, decrease quality of life, and increase psychosocial distress [10]. Currently, the treatment for DSPN chiefly includes diabetic management via tight glucose control and pain relief for neuropathy [11, 12]. Medications such as narcotic analgesics, tricyclic antidepressants, anticonvulsants, phenothiazines, nonsteroidal anti-inflammatory drugs, and opioids have all been used with limited success. Adverse effects such as drowsiness, lethargy, and unsteadiness are frequent [13].

In their search for more effective treatment strategies for DSPN and ways of slowing the overall progression of the disease, many patients in China seek help using traditional Chinese medicine (TCM). Chinese herbal medicine (CHM) is one part of TCM and has a long history of use in China. CHM treatments for diabetes, including DSPN, include both oral and topical medications, used alone or in combination with Western conventional medicine. Several randomized clinical trials have suggested that CHM can alleviate symptoms, improve motor conduction velocity (MCV) and sensory conduction velocity (SCV), and delay the progression of DSPN [14–17].

A meta-analysis of 18 randomized clinical trials including 1575 cases of DSPN demonstrated a beneficial effect of TCM on clinical symptoms. In that analysis, treatment groups showed significant differences from control groups, without any serious side effects (*P* < 0.01) [18]. However, a subsequent meta-analysis of ten randomized controlled studies established that there is insufficient evidence regarding the efficacy of CHM for the treatment of DSPN because of the pronounced clinical heterogeneity of the included studies and small sample sizes of the included trials [19].

TangBi Formula consists of five herbal granules, namely *Astragalus membranaceus* (Huangqi), cassia twig (Guizhi), *Ligusticum chuanxiong Hort* (Chuanxiong), *Radix Paeoniae Alba* (Baishao), and *Caulis Spatholobi* (Jixueteng), that have been used to treat DSPN at the Outpatient Department of Guang An Men Hospital of the China Academy of Traditional Chinese Medicine for nearly three decades. Relevant case reports have demonstrated that TangBi Formula alleviates the signs and symptoms of diabetic peripheral neuropathy (DPN) and improves NCV measured by electromyogram (EMG) [20].

For all of these reasons, the efficacy of TangBi Formula in patients with DSPN is worth investigating because of the pleiotropic effect. In the present study, we assumed that TangBi Formula in addition to conventional treatment may be more efficacious than conventional treatment with respect to the alleviation of the signs and symptoms of DSPN and improvement in NCV as indicated by EMGs. If successful, the study may provide an evidence-based complementary therapeutic approach to slow or prevent the clinical progression of DSPN in patients. In this protocol, we detail the overall study design and approach. This project was supported by the Special Scientific Research Fund of Traditional Chinese Medicine Profession of China (grant number 201507001-11), and it was registered with ClinicalTrials.gov (NCT03010241; registered on 2 January 2017).

## Methods/design

### Study objectives

**Fig. 1:**
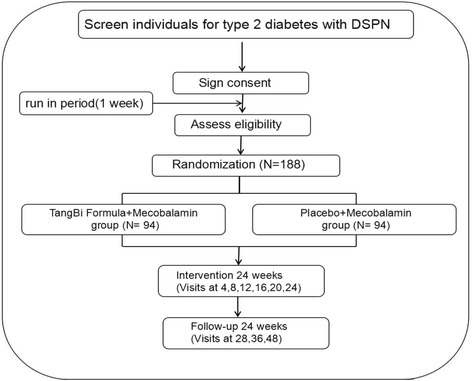
Entire design of this trial. *DSPN* Distal symmetric polyneuropathy

This clinical trial is designed as a multicenter, randomized, double-blind, placebo-controlled, parallel-group project. The trial flow is illustrated in Fig. 1. A superiority trial is planned to test the hypothesis that TangBi Formula used in combination with conventional treatment is more effective than conventional treatment alone in alleviating the clinical symptoms of DSPN and improving NCV of patients with DSPN. The treatment duration is set for 6 months.

### Design overview

The study design will integrate rigorous contemporary clinical research methodology in accordance with the principles set out in the Declaration of Helsinki and good clinical practice (GCP) guidelines according to the theory that guides the appropriate use of TCM in clinical practice. The rigorous design, organization, and conduct of the trial are supervised by a steering committee comprising two members from each participating center in addition to the chairman, scientific coordinator, and statistician. Six clinical centers in mainland China will participate in the trial, including Guang’anmen Hospital of the China Academy of Chinese Medical Sciences, the First Affiliated Hospital of Liaoning University of Traditional Chinese Medicine, the First Affiliated Hospital of Anhui University of Traditional Chinese Medicine, Hubei Hospital of Traditional Chinese Medicine, Zhengzhou City Hospital of Traditional Chinese Medicine, and Hebei Yiling Hospital. Data management and statistical analyses will be performed by independent data handlers and data analysts at the Institute of Basic Research in Clinical Medicine, China Academy of Chinese Medical Sciences. All of the patients must personally sign and date an informed consent document before randomization.

### Inclusion criteria

The inclusion criteria are outlined below.

1. Meet the diagnostic criteria of diabetic DSPN:
  - Have a history of type 2 diabetes.
    – Diagnosis of type 2 diabetes mellitus [21] is defined as fasting plasma glucose ≥ 126 mg/dl (7.0 mmol/L)
    Fasting is defined as no caloric intake for at least 8 h.*
    OR
    Two-hour plasma glucose ≥ 200 mg/dl (11.1 mmol/L) during an oral glucose tolerance test. The test should be performed as described by the World Health Organization using a glucose load containing the equivalent of 75 g of anhydrous glucose dissolved in water.*
    OR
    HbA1c ≥ 6.5% (48 mmol/mol). The test should be performed in a laboratory using a method that is National Glycohemoglobin Standardization Program-certified and standardized to the Diabetes Control and Complications Trial assay.*
    OR
    In patients with classic symptoms of hyperglycemia or hyperglycemic crisis, a random plasma glucose ≥ 200 mg/dl (11.1 mmol/L).*
    – Patients with type 2 diabetes and glycemia, effectively managed or not.
  (b). The main symptoms are numbness of the limb extremities; spontaneous pain (e.g., burning pain, tingling, dull pain); and paresthesia, usually distributed like gloves or socks, starting from the lower limbs, symmetrically; foot pain sense and diminished temperature sensation, vibration decreased or disappeared, but motor function basically intact.
  (c). EMG NCV decreases: We plan to diagnose patients with EMG NCV decreases when an EMG indicates that any of the 16 nerves of the bilateral motor nerves (median nerve, ulnar nerve, sural nerve, and superficial peroneal nerve) and the sensory nerves (median nerve, ulnar nerve, peroneal nerve, and tibial nerve) is conducting signals significantly lower than in healthy individuals.

2. The patient’s age is between 30 and 70 years.
3. The patient provides signed informed consent.

*In the absence of unequivocal hyperglycemia, results should be confirmed by repeat testing.

### Exclusion criteria

Exclusion criteria include the following:

1. Recent use of antioxidants, such as vitamin E or vitamin C; acute infection; liver or kidney dysfunction; acute complications of diabetes; severe cardiovascular and cerebrovascular disease; and neuropathy caused by long-term alcohol consumption or other factors.
2. Cardiovascular, liver, kidney, hematopoietic system, or other serious primary disease; serum transaminase more than double the normal value; serum creatinine (SCr) greater than the upper limit of normal; and psychiatric patients.
3. Pregnancy, preparation for pregnancy, or lactation, or any history of drug allergy.
4. Participation in another drug clinical trial or use of any other drug within the previous month.
5. Systolic blood pressure > 160 mmHg or diastolic blood pressure > 100 mmHg.
6. Having diabetic ketoacidosis, any other type of ketoacidosis, or severe infection within the previous month.
7. Any excessive consumption of alcohol or any consumption of psychoactive substances, drug abuse, or drug dependence during the past 5 years.
8. Any other disease or condition capable of reducing the possibility of entry or complicating the entry as determined by the researchers’ judgment, such as frequent changes in work environment, unstable living environment, or similar factors that could cause loss of contact.

### Conditions and procedures for withdrawal of study subjects

Subjects who become in eligible to continue participating in the study must be withdrawn.

### Researcher’s decision

The investigator may decide to remove subjects from the study for any of the following reasons:

1. During the study, the subject may experience some complications or physiological changes rendering him or her unsuitable for further participation.
2. During the study, the subject may be poorly compliant, such as failure to take at least 80% and no more than 120% of the prescribed amount of medicine.
3. Any breaking of blindedness or emergency unsealing of patient information.
4. Any use of drugs prohibited by the study plan during the study period.

### Subjects’ voluntary withdrawal

As stipulated in their informed consent forms, the subjects have the right to withdraw from the study at any time. Participants who do not formally withdraw from the study but cease to accept medication and undergo testing or who become impossible to contact are also considered withdrawn. The researcher should realize and record the reasons for subjects’ withdrawal to the greatest extent possible, noting whether the treatment is obviously ineffective, difficult to tolerate owing to adverse reactions, or whether the patient cannot continue to participate in clinical research for some other reason, such as economic or personal factors. Whatever the reason, the case record should be retained; the last test results will be carried forward to the final result; and a full dataset will be analyzed for the treatment’s efficacy and adverse effects.

### Intervention measures

All patients in the group will be treated with conventional treatment, which includes oral mecobalamin (500-mg tablet three times per day), diet, exercise, and oral medicine, to ensure access to steady levels of blood glucose, blood lipids, and blood pressure according to American Diabetes Association guidelines. On this basis, subjects will be randomly assigned to receive placebo (6 g/bag two times per day) or TangBi Formula (6 g/bag two times per day) by the central randomization system using a randomly permuted block design with block sizes of two and four. Upon production, study medications will be packaged and transferred to numbered bottles by designated pharmacists according to the randomized list prepared by the trial statistician. The treatment will last for 6 months. All of the people involved in the trial (patients, investigators, project manager, data management team, clinical research associates, and statisticians) will be masked with respect to the group assignments. Outcome analyses will be performed by analysts blinded to the group assignments by use of a central randomization system in which the codes will be kept by an independent allocator and revealed only after treatment and analyses are completed.

### Data collection and monitoring

All data will be recorded by trained clinical investigators in a standardized case report form (CRF) and instantly recorded in the database via the ClinResearch Electronic Data Capture System at http://www.tcmcec.net/crivrs/. To ensure the accuracy and reliability of the data, the study monitor will verify and cross-check the CRFs against the investigator’s source document records and drug-dispensing log. Missing data or specific errors in the data will be detected by programs, and the results will be sent to the investigator for resolution. Except for the treatment code, any individual identification of the subjects will not be released until the database is closed. Written documentation of changes will be available via electronic logs and audit trails. Original CRFs will be kept at the participating center for 5 years after completion of the study. Three committees have been established to guarantee the validity and integrity of the intervention protocols by the multicenter trial coordination group. The first is the clinical trial guidance committee, which is responsible for study design and the implementation process. The second is the data and safety monitoring board, which will monitor the data collection process to control its quality. The third is the outcome evaluation committee, which will evaluate the key outcomes (including outcome measurements and adverse events [AEs]).

### Study procedures

**Fig. 2:**
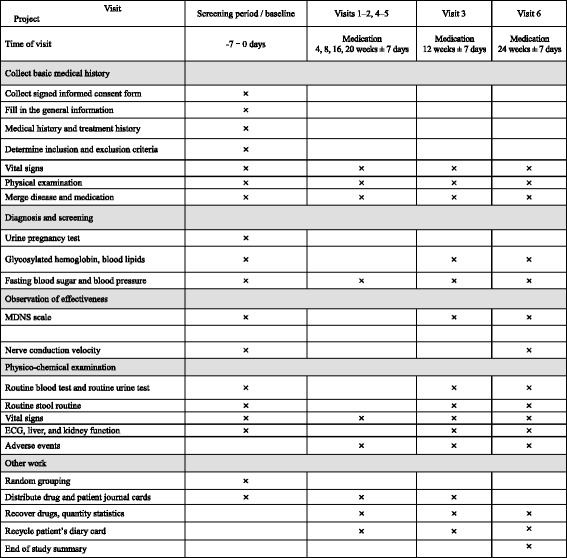
Treatment schedule. *MDNS* Michigan Diabetic Neuropathy Score

During the treatment, the following information will be collected and recorded: basic medical history, diagnosis and screening, observation of effectiveness, and physicochemical examination. Also, the following data will be recorded at visits 1–6: vital signs, physical examination, merge disease means comorbidity, fasting blood sugar, blood pressure, and AEs. In addition, glycosylated hemoglobin, blood lipids, Michigan Diabetic Neuropathy Score (MDNS), Electrocardiogram (ECG), and liver and kidney function will be assessed at visit 3, and glycosylated hemoglobin, blood lipids, MDNS, ECG, liver and kidney function, and NCV will be added at visit 6. All information related to the treatment schedule is provided in Fig. 2.

### Ethical issues

This study has been approved by the ethics committee of Guang’anmen Hospital of the China Academy of Chinese Medical Sciences (number 2016-096-KY-01). Each participating center obtained approval from its local institutional review board. All of the study participants will provide written informed consent prior to participation.

### Randomization and allocation

Treatment allocation occurs when the study participant meets the inclusion criteria and signs the informed consent form. The therapist will then register the participant in the database, which in turn asks if the participant is willing to be randomized. After the therapist enters “yes,” the specific randomization program behind the form displays the participant’s group assignment number (placebo versus Chinese medical herb). Specific randomization lists will be computer-generated and concealed from the researchers by a senior data manager not involved in the study. This information will remain confidential and will not be made available to the investigator. The allocation list will be protected by password-accessible files and held by an independent noninvestigator. In the event of a medical emergency, the individual’s randomization code and group allocation can be identified.

### Sample size

According to the preliminary study of TangBi prescription data, the placebo group scale (MDNS) score difference was 3.45 and the TangBi prescription scale score was 4.14, with an SD of 1.33. The sample size was estimated using a hypothesis test formula of measurement data: *N* = 2 × [(Zα + Zβ) × δ/*d*]^2^, where *N* is the sample size and δ is the estimated SD, *d* is two groups’ continuous variable mean difference, and Zα and Zβ are the corresponding standard normal differences. Supposing α = 0.05 and β = 0.10, according to the one-sided check table, the quantile Zα = 1.64485 and Zβ = 0.84162, and these are entered into the formula. It is estimated that a sample size of 78 subjects per group will be required, taking into account 20% dropout and withdrawal. This results in 188 participants in total. Intention-to-treat analysis will be used to minimize bias due to dropouts.

### Bias analysis

The evaluation index of this study is clinical symptoms, affected by many factors. The following factors may affect or bias the results: (1) blood glucose levels, (2) existing treatment measures, and (3) the conditions under which EMG is determined. These influencing factors are addressed as follows. For factors influencing blood glucose levels, a randomized, double-blind study design will be used to ensure consistency across the two groups. Administration of methycobal can reduce the psychological effect on patients and increase patient compliance. EMG specialists should be trained consistently, and the standards for fixed EMG should be established as a standard operating procedure for this study.

### Outcome measurements

#### Primary outcome measures

1. Changes in clinical signs and symptoms in patients with DSPN: Changes in clinical signs and symptoms will be evaluated prior to treatment and at weeks 12 and 24 of the treatment phase.
2. Changes in the MDNS are compared between the two groups before and after medication. The clinical signs and symptoms of patients will be recorded using the MDNS. MDNS will be evaluated prior to treatment and at weeks 12 and 24 of the treatment period.

#### Secondary outcome measures

1. Changes in NCV: The changes in NCV of each nerve will be compared before and after administration of medicine. Changes in NCV will be evaluated prior to treatment and at week 24 of the treatment period.

Each center has a designated EMG specialist to ensure the accuracy of EMG reading. Electromyographic detection is to be performed using Neurocare-C electromyography (Neurocare Center Co., Shanghai, China) and evoked potential instruments to determine MCV and SCV of the bilateral median nerve, ulnar nerve, common peroneal nerve, and tibial nerve. The room should be kept quiet, and the temperature should remain at about 25 °C. Skin temperature must remain between 28 °C and 30 °C, and hyperthermic treatment should be provided to patients with low body surface temperature. SCV is determined using the reverse method, which means proximal stimulus and remote reception. The sensory waveform is derived from the average deconvolution by machine automatically. SCV value is the measured distance (between receiving point and the stimulus point)/incubation. MCV is determined in the corresponding distal muscles. The corresponding nerve branch is superstimulated to elicit two action potentials: far end and near end. MCV is the distance between two points/difference in incubation between the two points. The NCV normal value refers to the clinical electromyography standard for the median nerve and ulnar nerve with MCV ≥ 45 m/second and SCV normal values ≥ 47 m/second individually. MCV normal values of peripheral nerve and tibial nerve separately were ≥ 42 m/second and ≥ 40 m/second. Values below those stipulated above indicate that NCV has slowed down, that means NCV abnomal.

2. Changes in clinical signs and symptoms: The clinical symptoms and signs of the patients will be compared before and after taking the medicine and will be evaluated prior to treatment and at weeks 12 and 24 of the treatment phase.

### Safety assessment and adverse events

**Fig. 3:**
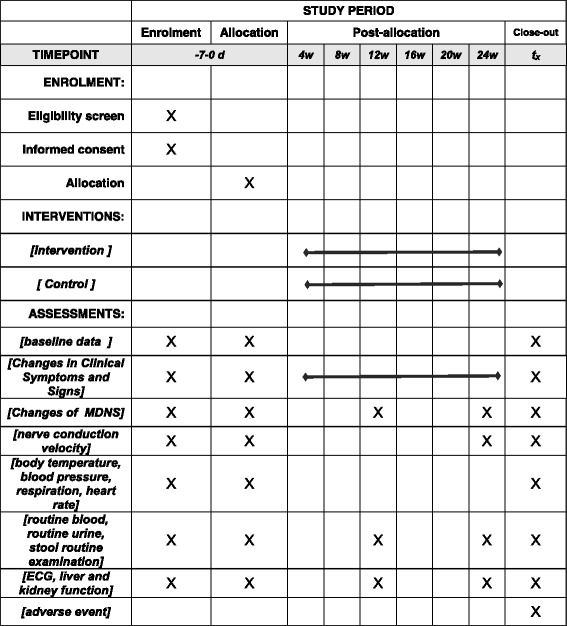
Content for the schedule of enrollment, interventions, and assessments

Safety will be monitored for 6 months after the intervention and evaluated on the basis of the incidence of AEs, including clinically significant changes in physical examinations, vital signs, and standard clinical laboratory tests. Vital signs include body temperature, blood pressure, respiration, heart rate, and similar factors at months 0–6 Routine blood examination, routine urinalysis, and routine stool examination will be done at months 0, 3, and 6. ECG, five items assessing liver function (alanine aminotransferase, aspartate aminotransferase, γ-glutamyl transpeptidase, alkaline phosphatase, and total bilirubin), renal function (blood urea nitrogen), and SCr will be measured at months 0, 3, and 6. In addition, we will make at least one on-site monitoring visit during the study to ensure that each participating center complies with the study protocol and GCP principles. AEs, such as signs and symptoms and other ailments, will be documented at every study visit. Each AE will be classified as a mild, moderate, or severe AE, and its correlation with the intervention drugs will be assessed. Severe AEs will be reported to the principal investigator and the ethics committee within 24 h. The data and AEs will be accurately and appropriately recorded on the CRF. All of the AEs will be recorded, monitored, and treated until properly resolved. The schedule of enrollment, interventions, and assessments is presented in Fig. 3.

### Statistical analysis

Three analysis sets will be used for analyzing data. First is the full analysis set (FAS), which refers to the ideal set of subjects as close as possible to the principle of intentionality analysis (including all of the subjects randomized to enroll and receive at least one treatment). If any given case is missing a main variable, the results of the closest observation are carried forward to the absence of the test data. The number of subjects evaluated at the end of each group is kept as consistent as possible before the start of the study. Second is the per-protocol set (PPS), in which all of the factors are in accordance with the research plan; patients show good compliance, using 80–120% of the prescribed dose; the CRF contains the details of the case; and the main variables can be determined. All of the baseline variables must be available, and there must be no major violation of the test plan. Third is the safety analysis set (SS), in which all of the subjects must receive at least one treatment after randomization.

Demographic and other baseline characteristics and efficacy analysis are set as the FAS and PPS. Safety analysis procedures are set as the SS. Data will be collected and analyzed using SAS 9.3 software (SAS Institute, Cary, NC, USA), and statistical analysis will be completed by the third-party personnel statisticians. All of the statistical tests are two-sided, and statistical significance is set at *P* ≤ 0.05.

### Statistical analysis method

#### Selection and completion

Cases were selected from among those who completed the study and those who dropped out. The selected cases and number of analytical datasets are described for safety and effectiveness. Cases not selected for PPS will also be listed.

#### Baseline equalization analysis descriptions

Baseline was defined as 0 days (when subjects were enrolled). The equilibrium analysis of basic values is focused on the basic demographic characteristics, vital signs, and indices related to curative effect to demonstrate whether the two groups of basic conditions are comparable. According to data distribution, the *t* test or Wilcoxon rank-sum test will be applied to compare the quantitative index of the two groups (such as ages, disease courses, blood glucose, glycohemoglobin [GHb]) by listing the number of cases, mean, SD, median, maximum, and minimum. The chi-square test and Fisher’s exact test are used to compare the qualitative index (such as gender, nationality, marital status, heart rate (HR), past history, family history, allergy history, previous treatment) by listing frequency and percentage.

#### Comparison of factors affecting test evaluation

For analysis of concurrent medication, the number and percentage of cases will be listed and compared using the chi-square test and Fisher’s exact probability method. For analysis of medication compliance, the number of noncompliant cases, those taking < 80% or > 120% of the stipulated dose, and compliant cases, those taking 80–120% of the stipulated dose, and the percentage of each are to be listed and compared using the chi-square test and Fisher’s exact probability method.

The medication period is the time of final medication minus the time of first medication plus 1 day. The number of cases, mean, SD, median, maximum, and minimum will be calculated and compared using the chi-square test and Fisher’s exact probability method.

#### Effectiveness analysis

For the main endpoint of curative effect, the MDNS and its changes relative to baseline after treatment are described. The number of cases, mean, SD, median, maximum, and minimum will be calculated and compared within each group using a paired *t* test or the Wilcoxon signed-rank test. Changes relative to baseline after treatment will be compared between groups using the *t* test or the Wilcoxon rank-sum test.

### Secondary index of curative effect

The changes in NCV, symptoms, and their differences relative to baseline will be described. The number of cases, mean, SD, median, maximum, and minimum will be calculated and compared within each group using the *t* test or the Wilcoxon signed-rank test. Changes relative to baseline after treatment will be compared between groups by *t* test or Wilcoxon rank-sum test.

### Monitoring index

The indices of FBG, GHb, blood pressure (BP), and lipids and their changes relative to baseline are described. In addition, the number of cases, mean, SD, median, maximum, and minimum will be calculated and compared in groups by *t* test and signed-rank test. Changes relative to the baseline after treatment will be compared between groups by *t* test and Wilcoxon rank-sum test.

## Discussion

DSPN remains a major public health problem. The aim of DSPN treatment is to decrease symptoms and reduce the risk of hospital admission, raise quality-of-life standards, and reduce the incidence of disability. Recently, the approach to therapy has been focused on symptomatic relief. TCM has a history of use in China going back several thousand years. Some types of TCM therapy, such as Tang-tong-fang, have been shown to be beneficial in patients with DPN, a common complication secondary to diabetic microvascular injury [22]. Reports have shown that TCM has had some success in the treatment of DPN. Some studies concentrate on nerve repair and regeneration, signaling pathways, neurotrophic factor, free radicals, and so forth [23–27], but there is less clinical research on the interaction of DSPN with TCM. We designed this study for optimal transparency and minimal reporting bias to improve the standardization of data collection (Additional file 1).

### Trial status

Patient recruitment began in March 2017. At the time of manuscript submission, 73 patients had been recruited, and no subject had yet completed the treatment.

## Additional file

Additional file 1: SPIRIT 2013. (DOC 124 kb)

## Abbreviations

AE: Adverse event
CHM: Chinese herbal medicine
CRF: Case report form
DSPN: Distal symmetric polyneuropathy
EMG: Electromyogram
FAS: Full analysis set
GCP: Good clinical practice
GHb: Glycohemoglobin
MCV: Motor conduction velocity
MDNS: Michigan Diabetic Neuropathy Score
PPS: Per-protocol set
SCr: Serum creatinine
SCV: Sensory conduction velocity
SS: Safety analysis set
TCM: Traditional Chinese medicine
